# Design-with-Nature for Multifunctional Landscapes: Environmental Benefits and Social Barriers in Community Development

**DOI:** 10.3390/ijerph10115433

**Published:** 2013-10-28

**Authors:** Bo Yang, Ming-Han Li, Shujuan Li

**Affiliations:** 1 Department of Landscape Architecture and Environmental Planning, Utah State University, 4005 Old Main Hill, Logan, UT 84322, USA; E-Mail: shujuan.li@usu.edu; 2 Department of Landscape Architecture and Urban Planning, College of Architecture, Texas A&M University, College Station, TX 77843, USA; E-Mail: minghan@tamu.edu

**Keywords:** environmental planning, stormwater management, interdisciplinary design, landscape performance, landscape preference, GIS

## Abstract

Since the early 1970s, Ian McHarg’s design-with-nature concept has been inspiring landscape architects, community and regional planners, and liked-minded professionals to create designs that take advantage of ecosystem services and promote environmental and public health. This study bridges the gap in the literature that has resulted from a lack of empirical examinations on the multiple performance benefits derived through design-with-nature and the under-investigated social aspect emanated from McHarg’s Ecological Determinism design approach. The Woodlands, TX, USA, an ecologically designed community development under McHarg’s approach, is compared with two adjacent communities that follow the conventional design approach. Using national environmental databases and multiple-year residents’ survey information, this study assesses three landscape performance metrics of McHarg’s approach: stormwater runoff, urban heat island effect, and social acceptance. Geographic Information Systems (GIS) was used to assess the development extent and land surface temperature distribution. Results show that McHarg’s approach demonstrates benefits in reducing runoff and urban heat island effect, whereas it confronts challenges with the general acceptance of manicured landscapes and thus results in a low safety perception level when residents interact with naturally designed landscapes. The authors argue that design-with-nature warrants multifunctionality because of its intrinsic interdisciplinary approach. Moreover, education and dissemination of successful examples can achieve a greater level of awareness among the public and further promote multifunctional design for landscape sustainability.

## 1. Introduction

Multifunctional landscapes, by definition, are designed for multidimensional benefits [1,2,3,4]. Landscape architects, architects, and planners are charged with designing landscapes that meet diverse human needs, while also facilitating ecosystem functions [5,6]. The level of success in creating high-performance, multifunctional landscapes is attributed to the extent of integrating ecosystems’ metabolism into the design process [7,8,9,10]. In other words, the creation and maintenance of healthy human settlements share the same principle of that of healthy ecosystems. 

A multifunctional landscape design solution must embrace the various ecosystem services that have already been bequeathed to a land area. These services include (1) supporting and biophysical services (e.g., protecting and enhancing biodiversity and water quantity and quality); (2) provisioning services (e.g., production of energy and other utilitarian resources); (3) regulating services (e.g., waste reduction and reuse); and (4) cultural and social services (e.g., visual quality, beauty, human health, and recreational opportunity) [11,12]. These services, coupled with human intervention, (re)shape the natural and built environments for achieving project sustainability goals and better human well-being [13]. Many past studies on ecosystem services have focused on ad hoc evaluation of landscape structure, composition, and services using empirical evaluation or predictive models. Few studies have tackled ecosystem services from the perspective of how landscape *design* can contribute to these services. 

Although this study is certainly not the first attempt of this kind, it contributes to the literature by evaluating the design-with-nature concept experimented with in a large-scale community development project, in which landscape design and management regimen changed over time due to social barriers. American landscape architect and ecological planner Ian McHarg (1920–2001) first proposed the concept design-with-nature [14]. McHarg’s concept is deeply grounded in ecosystem services which promise to achieve multifunctional performance. But a better understanding of the efficacy of his design approach remains lacking [15]. 

## 2. Review of Relevant Literature

### 2.1. Ecosystem Services, High-Performing Landscape Design, and Human Well-Being

Although the consensus has not been reached regarding the concept of ecosystem services, a central theme of the discussion is how ecosystem functions, directly or indirectly, contribute to human well-being [16,17,18,19,20]. These services include not only the provision or maintenance of goods and benefits (e.g., air, food, and water) that meet basic human needs [21,22,23], but also their influences on people’s satisfaction with the environment, human psychophysiological stress reduction, and quality of life [24,25]. 

Conceptual frameworks that link ecosystem services and human well-being have been proposed [26,27,28,29]. A noteworthy one is the Millennium Ecosystem Assessment, which assessed global ecosystem changes and their impacts on human well-being [19,23,30,31]. Lu and Li [32] developed criteria and modeling framework for ecosystem health measurements, and Ash *et al.* [33] provided a guidebook for practitioners on assessing ecosystem services, performance, and human well-being. Another recent work by Tzoulas *et al*. [34] provided a comprehensive framework that shows the linkages and interactions among ecosystem services, human health, and the design of green infrastructure (e.g., green space).

Past studies have also quantitatively examined the influences of ecosystem services on environmental quality and human well-being. For example, Tratalos *et al*. [35] examined the relationships between urban form and several ecosystem performance metrics (e.g., stormwater runoff, maximum temperature, and carbon sequestration), and they concluded that at any given land development density, substantive opportunities exist to maximize ecosystem services. Other studies have evaluated people’s perceptions of environment quality and other ecosystem performance metrics using various methods such as scenario analysis [31,36], survey [37,38], and experimental analysis [25,39]. 

However, at least three critical challenges exist in regard to how design can achieve high-performing landscapes to facilitate ecosystem services. First, it is difficult to define landscape performance benchmarks because ecosystems are dynamic and constantly changing [40,41]. The services that designed landscapes can provide are partly dependent on the overall health of the ecosystem in which they operate [42,43], and contextual factors, such as land cover and urban development conditions, affect landscape performance. For instance, in dense urban environments, stormwater infiltration design is hindered by various factors such as soil sealing and subsurface constraints [44,45]. 

Second, in the assessment of landscape function interactions, challenges have emerged in trade-off analyses [46,47]. Multifunctional landscape design can yield both positive and negative effects on service provision, particularly social outcomes. Findings from community green spaces are mixed, for example. Green spaces can provide multiple benefits to residents (e.g., better sense of place and more interactions with neighbors) [48,49,50]; whereas unkempt or unmanaged ones may negatively affect residents’ perception of safety and, as a result, decrease their well-being [24,51,52]. 

Third, there is a lack of understanding of how spatial and temporal scales affect multifunctional designs [53]. Studies show that the positive benefits of green space cannot be generalized, because different types and configurations of green space may yield positive or negative health outcomes [54,55]. Additionally, long-term monitoring studies are greatly needed to assess urban green space performance and for informed design amendments that meet local sustainability goals [56]. 

### 2.2. Design-with-Nature for Multifunctional Landscape Design

One of the first designers who charted a comprehensive way of multifunctional landscape design and planning was Ian McHarg. In his benchmark book *Design with Nature* [14], McHarg presents an ecological design concept that is based on ecosystem services, and he lays the foundation for using ecological science as the base for design and decision making. From a regional environmental planning standpoint, multifunctional landscape design has become an emerging paradigm that tackles critical societal challenges such as population growth, scarcity of resources, environment degradation, and social equity [6,56,57,58,59]. 

McHarg’s design-with-nature concept is a precursor of the multifunctional landscapes design paradigm [57]. McHarg focuses on the natural, social, and cultural processes and sees design as an iterative process that is largely shaped by the interactions between humans and ecosystems. Simply put, design should let nature perform the maximum amount of work [14,60,61]. McHarg’s followers build upon his environmental focus and further strengthen social, economic, aesthetics, and public health dimensions of sustainability, and advance theoretical and learning frameworks, such as Lyle’s regenerative design [62], Nassauer’s “cues to care” [63,64], Johnson and Hill’s and Steiner’s frameworks for ecology and design [58,65], and Musacchio’s six *Es* for landscape sustainability [6,66]. 

Design for multifunctional landscapes requires a suite of well-targeted metrics that can evaluate project performance or success [11,57,67]. Current sustainable design evaluation systems rate project performance from a multifunctional perspective, such as in the Leadership in Energy and Environmental Design (LEED) [68] and the Sustainable Sites Initiative (SITES^TM^) [69]. A series of quantitative measurements (e.g., water, vegetation, and material selection) assess project performance and rate the project success in a tiered rating system. More recently, the Landscape Architecture Foundation launched the Landscape Performance Series to evaluate landscape projects’ performance, using more than 30 metrics (e.g., crime prevention, and public health and safety) [70]. 

Increasing evidence has shown that *design* can achieve multifunctional benefits should the role that *nature* plays is taken into consideration; that is, design-with-nature warrants multifunctional landscapes. For example, decentralized and naturalized ways of managing stormwater suggest benefits of runoff deduction and water quality enhancement [71,72,73]. Increasing tree canopy coverage mitigates urban heat island (UHI) effect and may reduce the incidence of heat-related diseases [74,75,76]. Additionally, to facilitate nature’s services it is important to tackle design problems from interdisciplinary perspectives. In more than ninety projects, McHarg called upon interdisciplinary professionals and the design process started with an inquiry of land intrinsic carrying capacity [60]. The design process respects, integrates, and facilitates multiple ecosystems’ processes, functions, and services. Anthropogenic uses or interventions shall become an integral part of, not a barrier to, these processes [7,42,60]. 

Past studies on landscape performance usually focus on individual performance metrics (e.g., stormwater) and at site-level scales (e.g., 50–100 acres) [77,78]. Studies that simultaneously examine projects’ multifunctionality have been few, and particularly rare in large-scale project settings [72,79]. A number of studies have accessed the environmental planning aspect of McHarg’s design approach [80,81,82,83]. However, his approach draws criticism partly because of the under-investigated social aspect (such as public acceptance of design), emanated from his Ecological Determinism approach [15]. The current study builds on previous investigations and further compares McHarg’s approach with conventional development approach by using empirical data. 

The objective of this study is to examine whether McHarg’s design concept is superior to conventional design concept in achieving multifunctional landscapes at large-scale community settings. The Woodlands, TX, USA (117 km^2^) is compared with two adjacent community developments that started around the same period, in the 1970s. As landscapes have reached their stable or equilibrium functioning stage after four decades of community development, it would be an appropriate point at which to conduct a comparative analysis that will provide insights into the landscape performance benchmarks. 

Three ecosystem services metrics are examined: stormwater runoff, UHI effect, and public acceptance of the design (e.g., safety perception). These metrics were chosen because they pertain to original design challenges in The Woodlands. Stormwater management is critical to the severe flood-prone site condition in Texas coastal region [84,85]. In addition to the potential stormwater benefits, preserving the natural vegetation is expected to mitigate the UHI effect during Houston’s summer months, when heat-related diseases and deaths have been a threat to human well-being [86]. Social barriers to McHarg’s design have been reported [87,88], and this study is among the first few that compare public acceptance of McHarg’s design with changes in landscape design using multiple years’ survey data. 

## 3. Materials and Methods

### 3.1. The Woodlands Project Background

The Woodlands is considered to be “the best example of ecologically based new town planning in the United States during the 1970s” ([89], p. 325). McHarg presided early phases of the design where he compiled a multidisciplinary project team that incorporated engineering, ecology, hydrology, meteorology, limnology, and plant ecology into the design process. McHarg’s design focused on stormwater management because of the flood-prone Texas coastal condition. His design is distinguished from the conventional community design in three aspects: (1) soil permeability is used to coordinate land-use type and development density; (2) more than 25% of the natural forest stand is preserved; and (3) open drainage is used instead of curb-and-gutter drainage [84,85,90,91]. The first aspect is a particularly unique land planning strategy. It is achieved by preserving land with high soil permeability (e.g., sandy soils) as open space and land with low soil permeability for development. Hence, runoff is infiltrated in close proximity to where it is generated [90]. 

Regional extreme storm events show the effectiveness of McHarg’s approach—The Woodlands survived a 100-year storm in 1979 and a 500-year storm in 1994 with little property damage, while Houston, located 50 km to the south, was severely flooded in both events [92,93]. In addition, studies have revealed that McHarg’s design was successfully used in the early phases [83,94]. However, the low public acceptance led to a shifting-back to the conventional approach (e.g., open drainage is visually unpleasant to average residents) [88,95]. A hybrid approach was used in the later phases of development. Curb-and-gutter drainage was used in later subdivisions, although open drainage was maintained in collector streets. Also, a higher percentage of manicured landscapes emerged in later community development [88,96]. Quite a few homeowners undermined the ecological concepts by cutting backyard trees and clearing shrubs to expand their manicured lawn areas [88,97]. 

**Figure 1:**
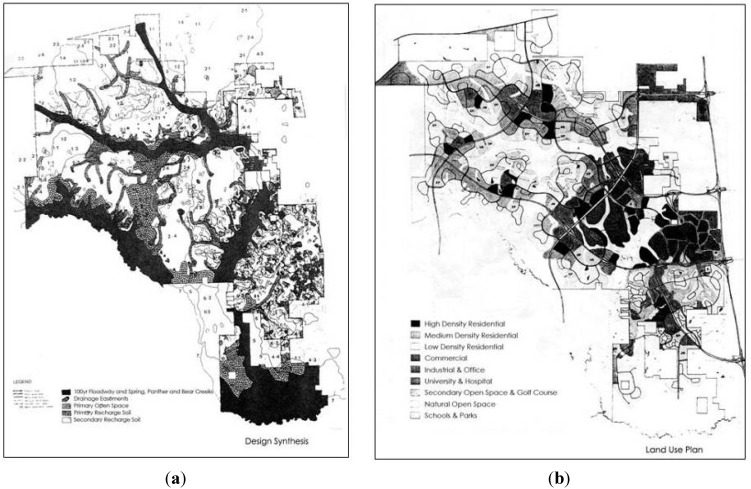
(**a**) Design synthesis ([85], p. 35); (**b**) Proposed land use plans ([85], p. 41) in The Woodlands. The proposed development locations are largely determined by soil patterns to allow maximum runoff infiltration (Image courtesy: WRT).

Figure 1 shows the design synthesis and land use plans according to McHarg’s original concept. Around 1985 The Woodlands deviated from these original plans and in 1997 the plans were almost abandoned [88,95]. 

### 3.2. Study Sites

**Figure 2:**
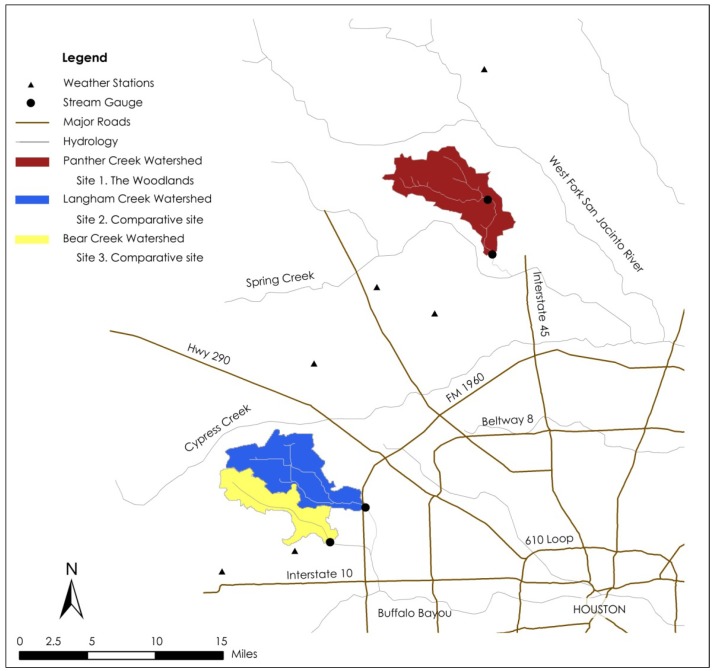
The Woodlands (Panther Creek watershed) and two comparative communities (Langham Creek and Bear Creek watersheds) in West Houston, TX, USA.

All three communities were started in the 1970s in suburban areas around Houston. This study uses watershed as the unit of analysis. Three watersheds that overlay the communities are delineated for comparison (Table 1, Figure 2). 

**Table 1:** Study sites and respective watersheds.

Watershed	Drainage area (km^2^)	Development start date	Population	Household number
1. Panther (Woodlands)	100.7	1974	66,143	24,655
2. Langham (comparative)	74.8	1978	56,976	16,973
3. Bear (comparative)	46.1	1976	33,763	9,559

Notes: Watersheds are defined by the U.S. Geological Survey gauging stations: No. 08068450 (Site 1), No. 08072760 (Site 2), and No. 08072730 (Site 3). Slopes in all the three watersheds are less than 1%. Population and household information is based on 2010 U.S. Census Block data.

**Figure 3:**
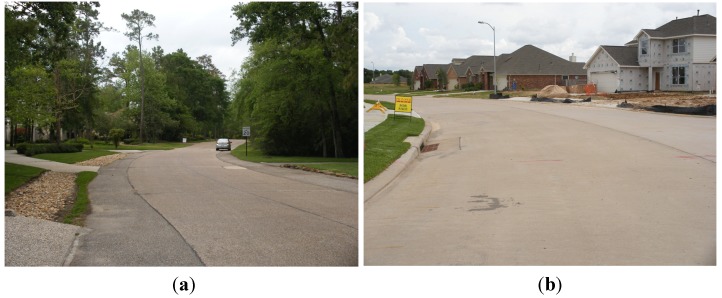
Typical neighborhood views in (**a**) The Woodlands (McHarg’s ecological design: curbless streets, open surface drainage, and well-preserved vegetation) and (**b**) comparative Houston communities (curb-and-gutter conventional drainage and less consideration of preserving vegetation).

Figure 3 shows typical views of the study sites, in respect to drainage and landscape designs. Panther Creek watershed (Site 1) consists of the majority of The Woodlands (Montgomery, TX, USA). Sites 2 and 3 contain conventionally developed communities in West Houston (Harris, TX, USA), falling within Langham Creek and Bear Creek watersheds, respectively. 

West Houston is a rapidly growing area. Its population has surpassed 1 million since 1999, and 34% of new single-family home construction in the Greater Houston area occur in west Houston [98]. Conventional “cookie-cutter” community design approach is used. Stormwater is managed through artificial curb-and-gutter system and natural vegetation is subjected to destruction during construction. Sites 2 and 3 are part of residential development areas, designated by the West Houston Association [98] and City of Houston General Plan [99]. 

### 3.3. Data Sources and Site Conditions

Table 2 provides data sources and explanations. Development, soil, precipitation, and streamflow data are used for stormwater runoff comparison. Landsat data are used for the UHI effect assessment. Residents’ survey data are used for public safety perception evaluation. Development extent and soil conditions were first assessed to demonstrate the background conditions of the comparative analyses.

**Table 2:** Data source and explanation.

Data	Source	Explanation
Land use land cover	NLCD website [100]	Provide development conditions of 2001 and 2006
Landsat	USGS Earth Resource Observation Systems Data Center website [101]	Used for land surface temperature estimation
Streamflow	USGS website [102]	Provide daily mean streamflow
Precipitation	NCDC website [103]	Provide daily precipitation
Soil	NRCS website [104]	Soil Survey Geographic (SSURGO) 1:24,000 scale
Resident survey	The Woodlands Township [105]	Seven years’ survey of Woodlands residents’ perception of safety when using park and community spaces

#### 3.3.1. Development Extent

Development data were used to quantify the impervious cover area. Imperviousness continues to be the single most important variable that defines the amount of urban development and predicts runoff volume [106,107]. It is also an essential variable to predict land surface temperature in the UHI assessment [75]. The latest development data were obtained from the US Geological Survey (USGS) 2006 National Land Cover Dataset (NLCD) at 30-m resolution [100]. Data accuracy of the NLCD national datasets (e.g., 1992, 2001 and 2006) ranges from 73% to 85% [108,109] and is regarded as acceptable in assessing land development and stormwater quantity and quality outputs [110,111]. 

For each land development pixel (30 × 30 m^2^), the two latest NLCD datasets (NLCD 2001 and NLCD 2006) contain information on percent developed imperviousness [112]. All the pixels that contain impervious cover were used to estimate the extent of development and the total impervious cover areas. Previous NLCD datasets (e.g., NLCD 1999) do not contain the same impervious cover information and were not included in this study. For the current study areas, NLCD 2001 dataset is in fact developed based on 1999 land use/land cover (LULC) conditions. Hence, 1999 LULC data (in lieu of 2001 LULC data) were used for the 1999 UHI effect assessment (described below) in order to match with the land development conditions. 

Geographic Information Systems (GIS) has been widely used in assessing the impact of land development and anthropogenic uses on the natural landscape [113,114]. In this study, GIS was used to quantify the total development area and further calculate the total impervious cover area. As aforementioned, the two latest NLCD datasets (*i.e.*, 2001 and 2006) process a GIS data layer that shows the percent developed imperviousness for each land development pixel (30 × 30 m^2^) [112]. For each watershed, totaling the developed pixels allows an estimation of the total development area, calculated with Equation (1) below:

Total developed area = 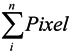
_developed_ × *900 m^2^*(1)
where *Total development area* represents the sum of areas where development has occurred (m^2^); *Pixel_developed_* represents each pixel that has impervious cover (e.g., developed); and 900 (m^2^) is the unit area of each pixel. 

As aforementioned, the NLCD 2001 and NLCD 2006 datasets have included the percent developed imperviousness for each pixel [112]. The total impervious cover area was then calculated by multiplying each individual pixel’s percent developed imperviousness with the unit area of each pixel (900 m^2^), calculated with Equation (2) below:

Total impervious cover area = 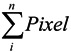
_developed_*× %Imperviousness × 900 m^2^*(2)
where *Total impervious cover area* represents the sum of areas that are classified as impervious (m^2^) (e.g., rooftop and road); *Pixel_developed_* represents each pixel that has impervious cover; % *Imperviousness* represents the percent of impervious cover in each pixel; and 900 (m^2^) is the unit area of each pixel. 

Table 3 shows the percent of developed land and percent of impervious cover area. It is evident that for both 2001 and 2006, Panther Creek watershed (Site 1) presents higher levels of impervious cover and development areas than Sites 2 and 3. In 2001, Site 1 imperviousness is around three times that of Site 2 and six times that of Site 3. In 2006, Site 1 imperviousness is approximately twice and three times of Site 2 and Site 3, respectively. More importantly, Site 1 imperviousness (31.8%) has surpasses the critical impervious cover threshold (c.a. 20–25%), after which much higher watershed runoff and erosion are expected [106], whereas impervious cover percentages in Sites 2 and 3 are still lower than this value. 

**Table 3:** Percent of developed land and percent of impervious cover areas in 2001 and 2006.

Site No.	Watershed	% developed land		% impervious cover	
		2001	2006	2001	2006
1	Panther creek (Woodlands)	62.2	70.9	27.1	31.8
2	Langham creek (comparative)	16.3	38.2	8.8	15.6
3	Bear creek (comparative)	15.8	36.9	4.6	12.0

#### 3.3.2. Hydrologic Soil Group Distribution

The soil dataset used was the 1:24,000 scale Soil Survey Geographic (SSURGO) database developed by the Natural Resources Conservation Service (NRCS). The U.S. Department of Agriculture (USDA) [115] defines four hydrological oil groups (A, B, C, and D) based on soil infiltration rates. A soils are sandy and loamy sand soils; B soils are sandy loam and loam soils; C soils are silt loam and sandy clay loam soils; and D soils are clay loam, silty clay loam, and clay soils. A soils have the highest infiltration rate, B and C soils have moderate infiltration rates, and D soils have the lowest infiltration rate. GIS was used to analyze the percentages of different hydrologic soil groups, which will provide insights into the overall stormwater infiltration capacity across the study sites.

**Figure 4:**
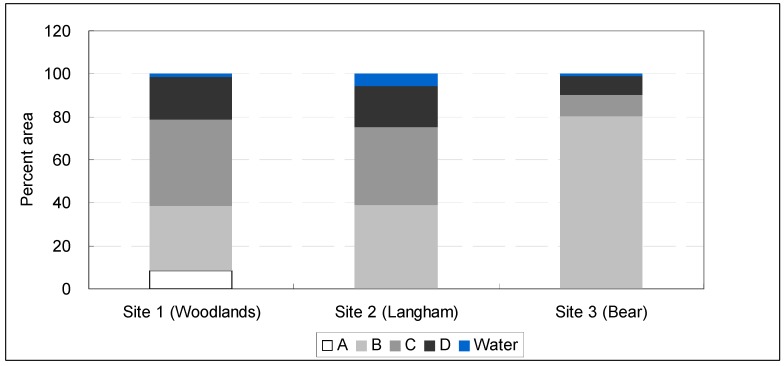
Area distribution of four hydrologic soil groups and water surface in Sites 1–3.

Figure 4 shows the area distribution of four hydrologic soil groups in the three watersheds. These four soil groups were further divided into two groups: A & B (sandy and loam), and C & D (silt and clay), in order to show the overall stormwater infiltration capacity (e.g., good *versus* poor). 

It is evident that stormwater infiltration capacity of Site 1 (The Woodlands) is lower than that of Sites 2 and 3, because Site 1 has a lower percentage of A & B soils (39% in Site 1 *vs.* 40% and 80% in Sites 2 and 3, respectively). 

### 3.4. Ecosystem Services Performance Metrics

#### 3.4.1. Precipitation-Runoff Correlation

It is hypothesized that runoff discharge volume (or detention volume vise versa) from different drainage systems will be different, and The Woodlands’ integrated stormwater management system would yield lower discharges than the other two sites. In lieu of directly assessing runoff volume, a widely used method is to examine the correlation of daily precipitation and runoff [116]. As the three sites are geographically close to one another (e.g., similar precipitation), low precipitation-runoff correlation will indicate that the watershed is less sensitive to rainfalls and presents a robust or resilient condition. 

Observed streamflow data are collected from the USGS website, based on the USGS stream gauging stations at the outlets of the three watersheds (see Table 1 and Figure 2). Historical precipitation data that are coincident with flow data were obtained from the National Climatic Data Center (NCDC) website [103]. The Thiessen polygon method [117] was used to estimate precipitation for all three watersheds. Three weather stations (COOPID No. 411956, No. 419076, and No. 414300) were identified for Site 1, and three other stations (COOPID No. 412206, No. 414704, and No. 414313) were used for Sites 2 and 3. The area weighted percentage of each station was used to calculate the composite precipitation value. Sample days for which rainfall data are missing were excluded from analysis; no attempt was made to estimate the missing data. 

Daily mean precipitation and daily mean flow data of 2006–2010 were used, if the corresponding precipitation is greater than 0 mm. Precipitation data were further grouped into three categories: 0–6 mm, 6–35 mm, and >35 mm to represent small, moderate, and large rainfall events [116]. Pearson’s *r* values show the correlation relationships—the higher the *r* value the more sensitive the drainage system responses to rainfalls, suggesting a vulnerability to flooding. Particularly if this correlation is weak in large rainfall conditions, it shows that the drainage system successfully maintains a steady streamflow and less prone to flooding.

#### 3.4.2. UHI Effect

The UHI effect is commonly known as a phenomenon in which urban atmosphere and surfaces present higher temperatures than the non-urbanized surrounding areas [75]. The magnitude of atmospheric temperature elevation has significant implications for human health, energy use, and air quality [118,119]. Land surface temperature (*T_s_*) is often used to estimate the surface UHI intensity [120]. Estimation for *T_s_* requires LULC and the corresponding infrared information. LULC data (30-m resolution) for 1999 and 2006 were obtained from the national NLCD datasets [100]. Eighteen LULC classes from the NLCD datasets were associated with this study. They were further grouped to match the five LULC classes specified by Stathopoulou *et al*. [121], including urban/densely built, suburban/medium built, mixed urban area, rural area, and water surface. 

The infrared information is assessed using high spatial resolution (60-m) satellite images provided by the Landsat Enhanced Thematic Mapper (ETM+) sensor with Landsat 7 satellite. Landsat 7 thermal images were obtained from the USGS Earth Resource Observation Systems Data Center [101]. Due to technical errors of the scanner, data collected from 2003 onward (including 2006 data) are impaired. Despite these errors, data quality is still considered to be acceptable and these data have been used in past studies [120]. 

It takes 16 days for Landsat 7 to rescan a location. However, all three sites are located along the Gulf Coast of Mexico, where cloudy days are common. To evaluate the maximum intensity of UHI effect, summer days with clear atmospheric conditions are preferred. The best quality data for this study were available in September, 1999 and May, 2006, which were used. 

The surface UHI temperature (*T_s_*) was estimated based on the methodology developed by Stathopoulou and Cartalis, 2007 [122]. First, calibration is conducted for Landsat 7 ETM + image data through two steps [123]: (a) calculating the spectral radiance *L* (Wm^−2^·sr^−1^·μm^−1^) based on the digital number (DN) values of thermal band 6 with Equation (3) below:

*L* = 0.0370558 × DN + 3.2 (3)

and (b) computing the at-sensor brightness temperature (BT) using the spectral radiance *L* with Equation (4):

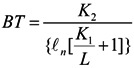
(4)
where *BT* is the at-sensor brightness temperature (Kelvin); *K_2_* is the calibration constant (1,282.71 K); *K_1_* is the calibration constant (666.09 Wm^−2^·sr^−1^·μm^−1^); and *L* is the spectral radiance at-sensor (Wm^−2^·sr^−1^·μm^−1^). Second, the land surface temperature (*T_s_*) is assessed after correction of emissivity for each LULC type [121]. The surface emissivity (*ε*) of the five composite LULC types are presented in Table 4. 

**Table 4:** Surface emissivity values by land cover type.

Land cover type	Emissivity
Urban/densely built	0.946
Suburban/medium built	0.964
Mixed urban area	0.950
Rural area	0.980
Water surface	0.990

*T_s_* is then calculated by Equation (5) below:

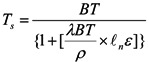
(5)
where *T_s_* is the land surface temperature (K); *BT* is the at-sensor brightness temperature (K); *λ* is the wavelength of emitted radiance (11.5 μm); *ρ* equals 1.438 × 10^4^ μm K; and *ε* is the spectral surface emissivity (see Table 3). Finally, GIS was used to map the *T_s_* of the three watersheds and adjacent areas for a summer month in 1999 and 2006.

#### 3.4.3. Safety Perception in the Woodlands

Starting in 1999, The Woodlands commissioned a third party survey company to conduct telephone interviews with the residents. These surveys solicit feedback on various community services (e.g., traffic, garbage collection, deed restrictions, *etc.*). Certain requirements were established to select interviewees, including that: (1) participants currently reside in one of the villages in The Woodlands; (2) participants need to be the head of household; (3) participants currently reside in a single family dwelling; (4) respondent/family/household members do not work in market research, advertising, or public relations, and (5) respondent/family/household members have never served on The Woodlands Township Board or been employed by any of The Woodlands Township Associations. 

One important survey question concerns residents’ safety perception in community in general and in community parks in specific. Seven survey studies have been conducted to date. The numbers of interviewees of each study are 575 (1999), 634 (2000), 727 (2002), 756 (2004), 941 (2005), 1,022 (2008), and 1,050 (2010). 

Residents’ safety perception was used as a surrogate for public acceptance of McHarg’s design. Because social barriers to McHarg’s design largely came from residents’ lack of appreciation of the naturalistic (or unkempt) appearance of landscapes [87,88], presumably the unkempt vegetation was the main reason for anxiety or fear of crime (e.g., low safety perception). Although safety perception is a very limited indicator of well-being, this longitudinal survey dataset would allow a valuable assessment of how residents’ appreciation of landscape design changed over time, particularly given the fact that The Woodlands deemphasized its design focus of vegetation preservation in the later phases.

The Woodlands resident survey data were obtained from The Woodlands Resident Study (see Table 2). These surveys consistently show a dichotomy between the early-built and later-built villages in respect to residents’ safety level perception [105]. For example, an excerpt from the 1999 The Woodlands Resident Study shows noteworthy differences in residents’ perception of safety in two subdivision villages: Grogan’s Mill and Alden Bridge. The former village followed McHarg’s design, whereas the latter shifted back to the conventional approach. Across the three community green space categories, the safety levels in Grogan’s Mill are significantly lower than those in Alden Bridge (*p* < 0.05). 

Hence, all the eight subdivision villages were divided into two groups to reflect the two different design approaches (McHarg’s *vs.* conventional). For each group and for each survey study year, the average score of the safety perception level was calculated with Equation (6):

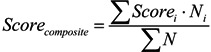
(6)
where *Score_composite_* represents the composite score of the safety level for the two subdivision village groups; *Score_i_* represents the score of the safety level in village *i*; and *N* represents the number of interviewees in village *i.*

There are no similar survey studies available for the Houston communities of Sites 2 and 3. Since The Woodlands shifted back to conventional landscape designs in the later phases (similar to those practiced in Houston), survey results from The Woodlands later-built villages would serve as a proxy for residents’ perception of conventionally design and managed community landscapes.

## 4. Results

### 4.1. Precipitation-Runoff Correlation

Similar annual precipitation amounts were observed in Sites 1–3, due to site proximity to one another. Table 5 shows the precipitation-runoff correlation expressed as Pearson’s *r* correlation coefficients*.* The higher the *r* value, the higher the correlation between precipitation and runoff is, and the more likely the site is subject to flooding. Despite the fact that Site 1 presents a much higher impervious cover percentage than Sites 2 and 3, its *r* value is lower than that of the latter two consistently across the three rainfall categories. 

**Table 5:** Regression analysis of precipitation and daily mean streamflow for 2006–2010.

Site No.	Watershed	Drainage method	Pearson’s correlation (*r*)			Avg. annual precip. (mm)
			0–6 mm	6–35 mm	>35 mm	
1	Panther creek (Woodlands)	Ecological	0.031	0.147	0.716	1.18 × 10^3^
2	Langham creek (comparative)	Conventional	0.046	0.341	0.814	1.19 × 10^3^
3	Bear creek (comparative)	Conventional	0.055	0.332	0.766	1.15 × 10^3^

To iterate that Site 1 impervious cover has exceeded a threshold (c.a. 20–25%) after which runoff is expected to increase substantially, if the conventional curb-and-gutter drainage method is used [106,124]. Given the fact that Site 1 has a lower percentage of recharging soils (e.g., A & B soils, see Figure 4) while a much higher percentage of impervious cover (31.8%), McHarg’s approach presents benefits of maintaining the original site hydrologic regime and mitigating flood. 

### 4.2. UHI Effect

**Figure 5:**
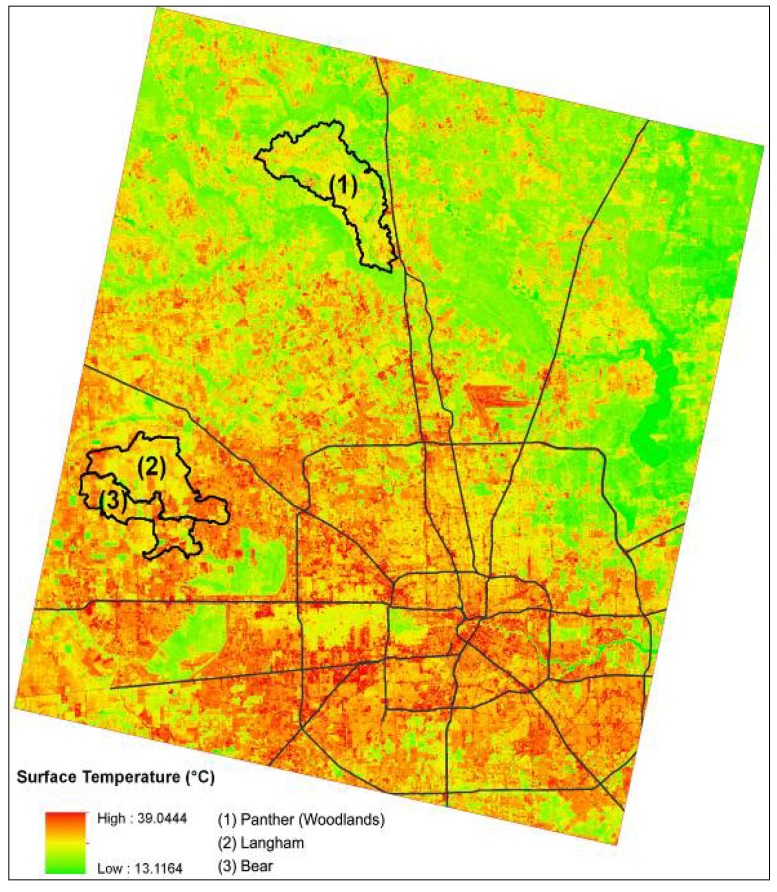
Surface temperature of Sites 1–3 and surrounding areas on 20 September 1999.

**Figure 6:**
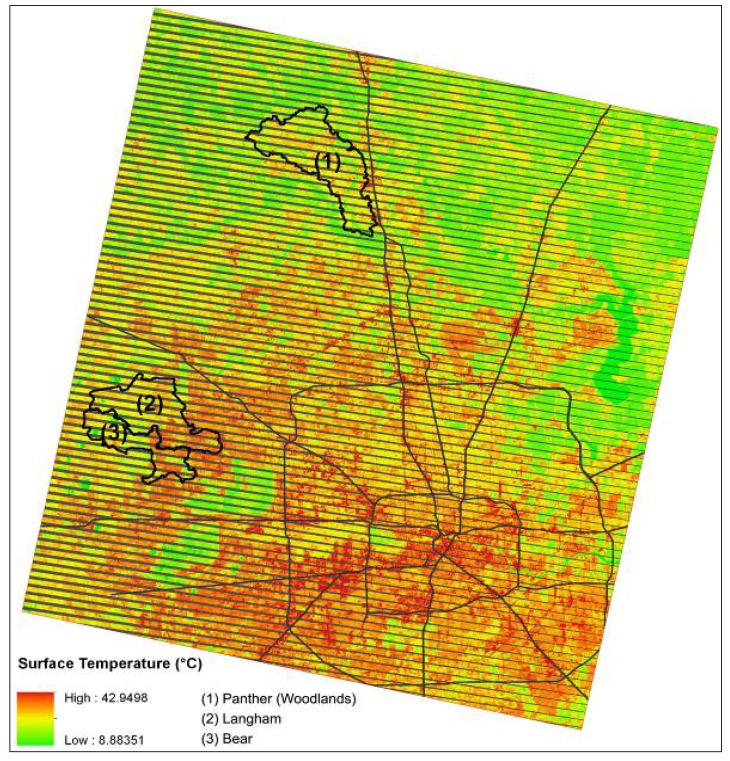
Surface temperature of Sites 1–3 and surrounding areas on 18 May 2006.

Figure 5 and Figure 6 show the land surface temperature (*T_s_*) distribution, and Table 6 shows comparisons of the mean surface temperatures of the three watersheds. It is evident that The Woodlands (Site 1) *T_s_* is lower than that of the conventionally developed sites (Sites 2 and 3) in both years examined. *Ts* in Sites 2 and 3 are 1.1–1.9 degrees (°C) higher than Site 1. Although Site 1’s development area and impervious coverage are much higher than Sites 2 and 3 (see Table 3), the extensively preserved forest land effectively mitigates the UHI effect by reducing the surface radiative properties (albedo) and ameliorates the ambient temperature. 

**Table 6:** Mean surface temperature (°C) on 20 September 1999, and 18 May 2006.

Site No.	Watershed	9/20/1999	5/18/2006
1	Panther creek (The Woodlands)	24.5	23.8
2	Langham creek (comparative)	26.3	25.5
3	Bear creek (comparative)	26.4	25.0

Preserving the original forest (tree canopy and understory) is mandated in The Woodlands Residential Development Standards [84,85,91,125]. However, this emphasis is typically lacking in Houston subdivision development Code of Ordinances [81]. In short, vegetation preservation as an important design strategy has shown benefits not only in hydrology, but also contributed to a better thermal environment. 

### 4.3. Safety Perception in the Woodlands

For each Woodlands survey study, an average score is calculated for residents’ perceived safety level in three community space categories. The results were further grouped into two subsets for comparison (the early- and later-built subdivision villages). The average scores are presented in Table 7. Across the three community space categories, residents express higher levels of safety perception in later-built villages. In contrast, in the early-built villages, residents express safety concerns [126]. The dense canopy and wild-looking understory invite feelings of being unsafe and of insecurity—the dense vegetation appears to be a hiding place for potential attackers and there is a fear of physical and/or sexual assault in this kind of environment [127,128]. 

**Table 7:** Summary of residents’ perception of safety on a 1–5 scale in The Woodlands (1 = Not safe, 5 = Very safe) from the past six resident studies.

Year	Planning method	In community parks	In neighborhood during day	In neighborhood at night
1999	Ecological	4.04	4.51	3.99
	Conventional	4.28	4.71	4.23
2002	Ecological	4.03	4.53	4.03
	Conventional	4.22	4.66	4.23
2004	Ecological	3.98	4.52	4.09
	Conventional	4.22	4.67	4.29
2005	Ecological	4.14	4.58	4.21
	Conventional	4.22	4.66	4.22
2008	Ecological	4.12	4.58	4.15
	Conventional	4.16	4.57	4.17
2010	Ecological	3.82	4.38	3.85
	Conventional	4.03	4.41	3.89

Notes: (1) For McHarg’s ecological design approach, four subdivision villages that fully or partially used his approach were involved in this calculation, including Grogan’s Mill, Panther Creek, Cochran’s Crossing, and Indian Springs. For the conventional approach, the other four subdivision villages were used for calculation, including Alden Bridge, College Park, Sterlling Ridge, and Creekside Park; (2) Conventional approach scored consistently higher than McHarg’s approach. The only exception is in 2008, under *in neighborhood during day*, McHarg’s approach scored slightly higher; (3) Year 2000 was excluded from this analysis because it used a different rating system and made it difficult to compare with other years’ scores.

In the later-built villages, more conventional landscape design and management approach is used. This approach allows manicured landscapes (e.g., lawn, annual flower beds) in community areas to fit average residents’ landscape preference. This approach may have affected residents’ safety perception over time, in that the gaps in scores between the two subdivision village groups (McHarg’s *vs.* conventional) decreased in recent years. 

## 5. Discussion

This study shows that McHarg’s design-with-nature concept can achieve performance benefits in stormwater runoff reduction and UHI effect remediation. Across the three rainfall intensity categories, correlation analyses show that when rainfall intensity increases, runoff volume does not necessarily increase in The Woodlands. The opposite is true in Houston communities. These results indicate higher volumes of runoff are expected from Houston communities than The Woodlands. Additionally, stormwater benefits may likely go beyond runoff reduction; additional benefits accrue, such as the reduced potential flooding cost and lower sedimentation control cost, as a result of reduction of runoff management. 

Additional benefits are likely to be true for the UHI effect mitigation. The results show that during summer months, The Woodlands’ land surface temperature can be almost 2 °C cooler than Houston communities. According to Adams [129], a 1.7 °C reduction in temperature can result in air quality benefits that are almost equal to replacing all the gas-powered vehicles with electric ones in a city. In addition to the expected air quality benefits, it is postulated that the 2 °C temperature drop in The Woodlands may yield other positive implications on human well-being, such as reduction in heat-related diseases [130], reduction in energy consumption [118], and culinary water consumption for landscape irrigation [131]. 

It is speculated that the ecosystem services go beyond stormwater reduction and UHI effect remediation. These services are partly maintained through the preservation of the natural stands of the pine forest [83]. The naturally vegetated open space and the extensive trail systems can provide various ecological (e.g., wildlife habitat), cultural and recreation (e.g., contemplation, environmental education, and wildlife watching), and healthy benefits (e.g., physical exercise opportunities and social interaction), and it is a low-maintenance solution [88,96,97,132].

Interesting, Table 7 shows that when the early-built villages of The Woodlands partly changed from McHarg’s design approach to more typical landscape designs as practiced in Houston communities (e.g., manicured lawn), the score of safety perception in the early-built villages increased. Also, the gaps in scores between the two subdivision village groups decreased (see Table 7). This indicates that landscape appearance plays an important role in influencing residents’ perceptions over time. Although there are no similar survey studies conducted in Sites 2 and 3, the results here seem to concur with the literature that highly maintained and managed landscapes contribute positively to good safety perception. 

The high performance, multifunctional landscape design is attributed to the multidisciplinary team that can integrate ecosystem services into the design process. For a project scale such as The Woodlands (117 km^2^), it is critical to draw expertise from multiple disciplines to better understand the site conditions and to solve complex design problems [60,133]. McHarg’s design focused on environmental aspect in the context of the Environmental Era and landscape preference and other aspects of human well-being were of less consideration. Although his ecological design approach presents challenges to the cultural preference of manicured landscape, some scholars argue that the natural beauty and aesthetics of wild urban nature are also of vital importance to quality of life [134,135]. In fact, there are possible design amendments that allow better acceptance of ecological design, e.g., to increase the visual penetration in The Woodlands open spaces to increase safety perception. When The Woodlands shifted back to the conventional approach of managing stormwater, flooding occurred, such as during a storm in 2000 and during Hurricane Ike in 2008, when the later phases of development suffered the most [93,136].

It is also important to note that there are some inherent challenges to design for both ecological benefits (e.g., from a wildlife perspective) and for human use [58]. In a park design, for instance, an irregular, voluminous shape is preferred for wildlife habitats, whereas for human use typically involves geometric and regular shapes. As Musacchio [137] pointed out in the Six *Es* theoretical framework for landscape sustainability, there needs to be an intricate balance between the environmental, social, and economic aspects to achieve sustainability goals. Every design has its main focus and project success should be measured based on its main project goal, rather than by a rigid set of metrics. 

Additionally, if the residents were convinced of the environmental benefits and the potential social, cultural, and economic benefits of McHarg’s design, public opinion on well-being in The Woodlands may be amended. For instance, after The Woodlands shifted to a conventional stormwater management approach, residents in later-built villages constantly complained about flooded streets and parks, whereas residents in early-built villages seldom have such complains [96]. 

Despite the promising environmental benefits, the study cannot address several issues and present limitations. For example, residents’ safety perception is a limited indicator of human well-being. There are other important indicators that are related to landscape design (e.g., access to green space, stress reduction, and perception of nature). Also, the dilapidation of park structures and amenities may contribute to safety concerns in the early-built villages. But these factors cannot be ruled out based on the available survey studies. The second-hand survey data allowed an indirect assessment of human well-being and quality of life. More refined survey studies (e.g., contingent valuation) are needed for future research, such as investigating residents’ willingness-to-pay for the ecosystem services provided by the ecologically designed landscapes. 

Moreover, using watershed as the unit of analysis cannot encompass all the study areas that are part of the land use plans. Around one third of The Woodlands’ early phases (e.g., followed McHarg’s design) do not lie in the Panther Creek watershed. Therefore, the efficacy of McHarg’s design may not be fully revealed because of the research design. Also, the study cannot completely tease out the performance of the early-built and later-built villages in respect to their ecosystem service performance because they were treated together as one study site. Further studies are also called for to examine the change of design approaches and the subsequent impact on ecosystem services. 

## 6. Conclusions

This study compares McHarg’s ecological design approach with the conventional approach being used in large-scale community developments. Using empirical data, the study demonstrated multiple environmental benefits on stromwater management and UHI effect mitigation that McHarg’s design-with-nature approach could bring. The study also revealed that residents’ perception of safety in wooded areas (e.g., community parks) was compromised, that is, wooded areas decreased the perceived safety. Such contrast findings raise a noteworthy question: do multifunctional high-performing landscapes always have positive performance on social, ecological/environmental, and health aspects? Or perhaps social and ecological needs may not always be in agreement, as shown in the findings of the study. 

McHarg’s design approach started in the 1970s, first originating from his seminal book *Design with Nature* [14]. In practice, it is evident that not only is sustainable design pertinent to environment stewardship; careful considerations also must be paid to human perceptions and cultural values, which (re)shape the way landscape is valued, appreciated, and managed. Landscape design needs to balance various considerations such as ecology, safety, and aesthetics. Sometimes those benefits are difficult to accomplish simultaneously. It is designers’ responsibility to demonstrate the performance benefits and educate the public to appreciate design-with-nature. 
